# Responses of LAI to rainfall explain contrasting sensitivities to carbon uptake between forest and non-forest ecosystems in Australia

**DOI:** 10.1038/s41598-017-11063-w

**Published:** 2017-09-15

**Authors:** Longhui Li, Ying-Ping Wang, Jason Beringer, Hao Shi, James Cleverly, Lei Cheng, Derek Eamus, Alfredo Huete, Lindsay Hutley, Xingjie Lu, Shilong Piao, Lu Zhang, Yongqiang Zhang, Qiang Yu

**Affiliations:** 1 School of Life Sciences, University of Technology Sydney, Sydney, Australia; 2 Key Laboratory of Vegetation Restoration and Management of Degraded Ecosystems, South China Botanical Garden, Chinese Academy of Sciences, Guangzhou, 510650 China; 3 China and CSIRO Oceans and Atmosphere, PMB 1, Aspendale, Victoria 3195 Australia; 4 School of Earth and Environment, the University of Western Australia, Crawley, Australia; 5 CSIRO, Land and Water, Canberra, Australia; 6 Research Institute for the Environment and Livelihoods, Charles Darwin University, Casuarina, Australia; 7 Peking University, Beijing, China; 8 State Key Laboratory of Soil Erosion and Dryland Farming on the Loess Plateau, Northwest A & F University, Yangling, 712100 China

## Abstract

Non-forest ecosystems (predominant in semi-arid and arid regions) contribute significantly to the increasing trend and interannual variation of land carbon uptake over the last three decades, yet the mechanisms are poorly understood. By analysing the flux measurements from 23 ecosystems in Australia, we found the the correlation between gross primary production (GPP) and ecosystem respiration (R_e_) was significant for non-forest ecosystems, but was not for forests. In non-forest ecosystems, both GPP and R_e_ increased with rainfall, and, consequently net ecosystem production (NEP) increased with rainfall. In forest ecosystems, GPP and R_e_ were insensitive to rainfall. Furthermore sensitivity of GPP to rainfall was dominated by the rainfall-driven variation of LAI rather GPP per unit LAI in non-forest ecosystems, which was not correctly reproduced by current land models, indicating that the mechanisms underlying the response of LAI to rainfall should be targeted for future model development.

## Introduction

**Figure 1:**
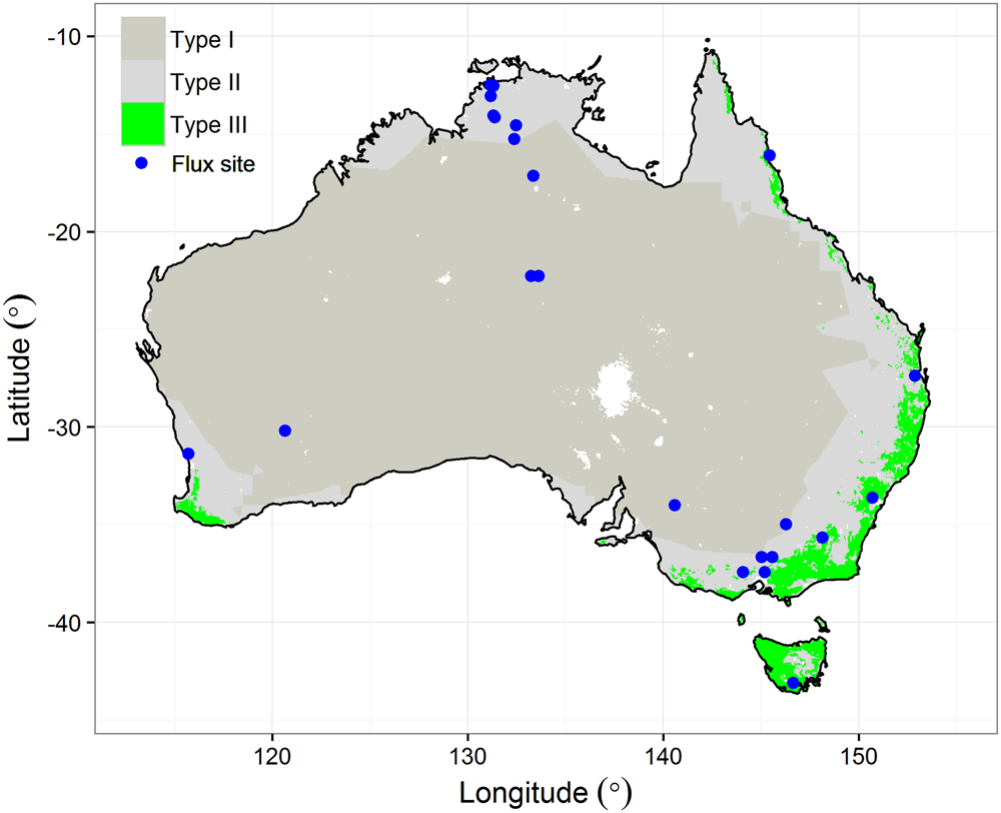
Distribution and definition of climate (Köppen-Geiger) and biome (IGBP land cover) space across Australia (total area = 7.56 × 10^6^ km^2^). Type I is non-forest and semi-arid ecosystems (77.6% of total area). Type II is non-forest and not semi-arid (18.8%). Type III is forested ecosystems and not semi-arid (3.5%). Combination of Type I and II is defined as non-forest ecosystems in our study. Solid points are locations of the 23 flux sites across Australia. Map was drawn using R version 3.2.4 (http://www.R-project.org/).

Recent studies have demonstrated that both the trend and inter-annual variation (IAV) of terrestrial carbon uptake over the past three decades were dominated by global non-forest (not covered with forest) ecosystems, and that Australian non-forest ecosystems (Types I and II as shown in Fig. 1 include grassland, savanna, woody savanna, shrubland and cropland) accounted for 57% of global terrestrial carbon uptake during the very wet year of 2010/2011^1,2^. These results are supported by remote sensing based estimates of vegetation biomass change over this period (estimated to be 0.05 Pg C year^−1^) in semi-arid savannas and shrublands of Australia and southern Africa^3^. Compared with temperate and tropical forest ecosystems in the world, these non-forest ecosystem generally are much less productive, and their significant contributions to both the trend and IAV of global terrestrial carbon uptake were unexpected. The underlying mechanisms driving this large contribution are not well resolved.

Net ecosystem production (NEP) is a small difference between two large fluxes, namely gross primary production (GPP) and ecosystem respiration (R_e_). Identifying the main drivers of these component carbon fluxes (GPP and R_e_) is critical for understanding the global carbon cycle, predicting future trajectories for atmospheric CO_2_ concentration and therefore climate change. At an inter-annual scale, variation of NEP substantially depends on the variability of climatic drivers and the different responses of GPP and R_e_ to those drivers^4^. GPP was found to be more sensitive to drought events than R_e_^5^. Globally, annual rainfall and mean annual temperature drive much of the inter-annual variability in GPP and R_e_^6–8^, particularly for non-forest ecosystems. For example decrease in rainfall after 2011 resulted in the the savanna ecosystems in central Australia switching from a strong sink to a weak source of C^9^. Furthermore, previous studies found that the net ecosystem carbon balance of Australian non-forest ecosystems was principally driven by year-to-year fluctuations in rainfall via changes in both ecosystem GPP and R_e_^10–12^.

Australia is the driest permanently inhabited continent on Earth, and is dominated by non-forest ecosystems. To identify the key mechanisms controlling the IAV of NEP for Australian terrestrial ecosystems, we analyzed carbon fluxes from 23 Australian eddy flux sites, which together covered all major Australian ecosystem types^13^. This study will identify the key mechanisms causing interannual variability in terrestrial ecosystem C balances of Australian forest and non-forest ecosystems, and assess whether those mechansims are correctly represented by some of the most advanced global land models. To achieve the second aim, we also compared the observed variances of log-transformed GPP, LAI, GPP/LAI and the covairnace between log-transformed LAI and GPP/LAI with the simulations from four process-based ecosystem models from the TRENDY (Trends in net land-atmosphere carbon exchange) compendium^14^.

## Results

### Contrasting sensitivities of non-forest and forest ecosystems to rainfall

**Figure 2:**
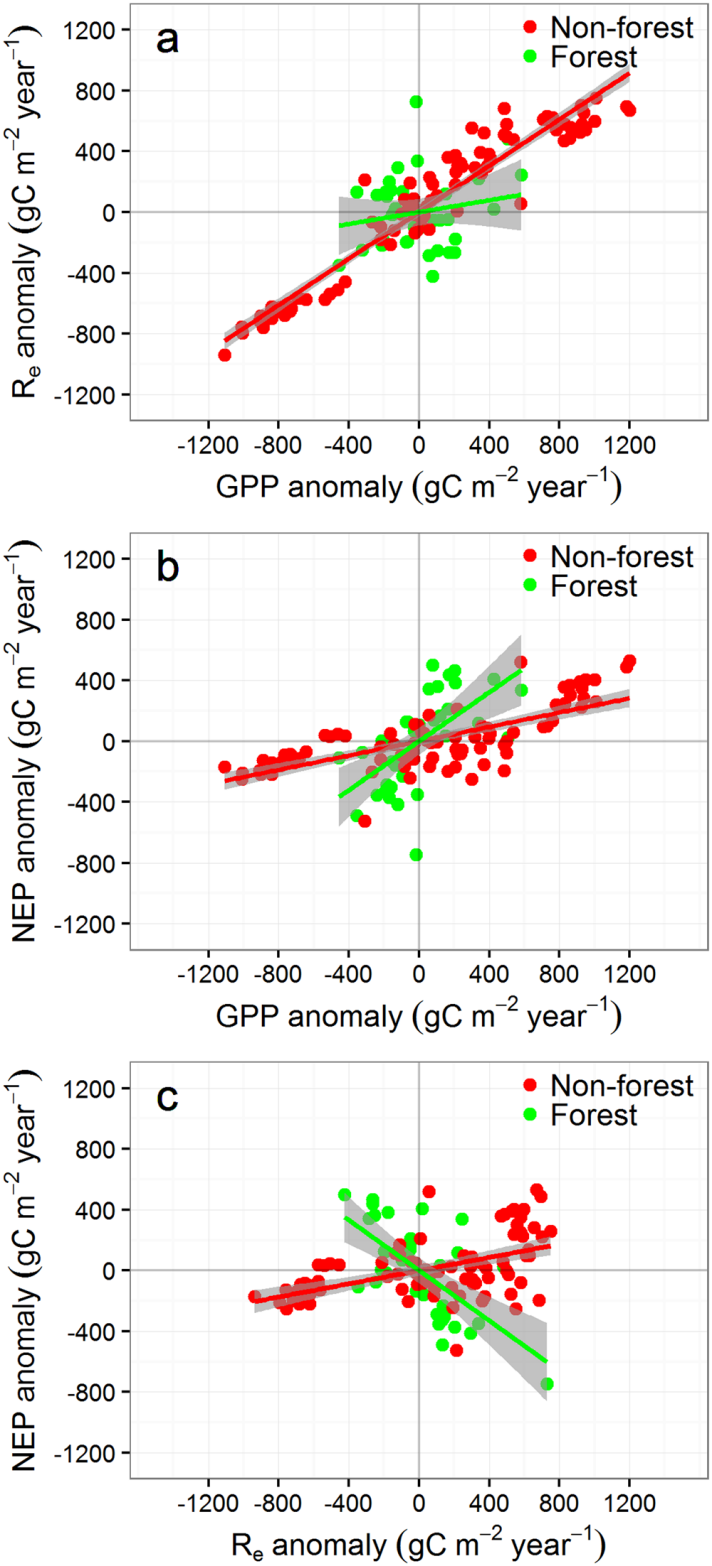
The relationships between annual anomalies of carbon flux anomalies from from the mean of all sites for each ecosystem type in Australia. (**a**) The correlation between gross primary production (GPP) and ecosystem respiration (R_e_). (**b**) The correlation between net ecosystem production (NEP) and GPP. (**c**) The correlation between NEP and R_e_. Anomalies were calculated as the annual fluxes minus the mean value of annual fluxes at all sites for each ecosystem type. Red and green solid circles denoted the flux anomalies for non-forest and forest ecosystems, respectively. The solid lines (red for non-forest and green for forest) are the best-fitted linear regression equations with the shaded area for 95% confidence intervals.

We first calculated the anomalies of annual flux variables (ΔGPP, ΔR_e_ and ΔNEP) by subtracting each annual mean value from the multi-annual mean of each ecosystem type (non-forest or forest ecosystem, see Table 1). The range of ΔGPP and ΔR_e_ anomalies of the non-forest ecosystems were 2.22 and 1.47 times larger than their respective values of forest ecosystems (Fig. 2a). This was despite the fact that the range in precipitation anomalies for non-forest (−800~1400 mm) was smaller thant that for forest (−1200~4000 mm). The linear correlation between ΔR_e_ and ΔGPP anomalies was significant for the non-forest ecosystems (ΔR_e_ = 0.76ΔGPP, *r* ^2^ = 0.93, *P* < 0.001), but not significant (we took *P* < 0.05 as significant) for the forest ecosystems (ΔR_e_ = 0.2ΔGPP, *r* ^2^ = 0.004, *P* = 0.29, Fig. 2a). ΔNEP were significantly and positively correlated with ΔGPP for both non-forest (ΔNEP = 0.24ΔGPP, *r* ^2^ = 0.55, *P* < 0.001) and forest (ΔNEP = 0.8ΔGPP, *r* ^2^ = 0.36, *P* < 0.001) ecosystems (Fig. 2b). ΔNEP was significantly and positively correlated with ΔR_e_ for non-forest ecosystems (ΔNEP = 0.22ΔR_e_, *r* ^2^ = 0.29, *P* < 0.001) but negatively correlated with ΔR_e_ for forest ecosystems (ΔNEP = −0.82ΔR_e_, *r* ^2^ = 0.42, *P* < 0.001) (Fig. 2c). Therefore GPP and R_e_ are positively correlated, and they together affect the internnual variation of NEP in non-forest ecosystems. However GPP and R_e_ are not significantly correlated, and they affect NEP independently in forest ecosystems (Fig. 2).

**Table 1:** Information about 23 eddy flux tower sites from OzFlux network (http://www.ozflux.org.au, see Beringer *et al*.^13^).

Site	Lon (°)	Lat (°)	*T*_mean_ range (°C)	*P*_rcp_ range (mm)	LAI (m^2^ m^−2^)	IGBP type	Ecosystem type	OP
Adelaide River	131.18	−13.08	26.7–26.9	1778–1935	1.04	SAV	Non-forest	2007–2008
Alice Springs	133.25	−22.28	21.7–24.3	143–415	0.30	WSA	Non-forest	2011–2013
Calperum	140.59	−34.00	17.3–18.8	211–511	0.44	OSH	Non-forest	2010–2016
Cow Bay	145.45	−16.10	23.5–24.5	2494–5566	4.18	EBF	Forest	2009–2015
Cumberland	150.72	−33.62	18.0–18.8	733–977	1.36	WSA	Non-forest	2013–2016
Daly Pasture	131.32	−14.06	24.4–26.0	1002–1704	1.50	GRA	Non-forest	2008–2012
Daly Uncleared	131.39	−14.16	25.7–27.6	759–1602	1.21	SAV	Non-forest	2007–2016
Dry River	132.37	−15.26	25.1–28.2	694–1449	1.16	SAV	Non-forest	2008–2012
Gingin	115.71	−31.38	17.3–20.0	525–667	0.89	WSA	Non-forest	2011–2015
GWW	120.65	−30.19	18.7–20.1	208–379	0.38	WSA	Non-forest	2013–2016
RDMF	132.48	−14.56	26.5–26.5	791–791	1.04	CRO	Non-forest	2012–2012
Riggs Creek	145.58	−36.65	15.0–16.1	92–552	1.26	GRA	Non-forest	2011–2014
Robson Creek	145.63	−17.12	19.1–19.7	2346–2387	4.53	EBF	Forest	2014–2015
Howard Springs	131.15	−12.50	25.7–28.3	813–2286	1.53	WSA	Non-forest	2001–2016
Samford	152.88	−27.39	18.9–19.7	672–1908	1.96	GRA	Non-forest	2010–2015
Sturt Plains	133.35	−17.15	24.2–27.8	404–992	0.49	GRA	Non-forest	2008–2016
Ti Tree	133.64	−22.29	23.0–23.7	366–674	0.32	WSA	Non-forest	2013–2016
Tumbarumba	148.15	−35.66	7.4–10.6	424–1502	4.17	EBF	Forest	2001–2015
Wallaby Creek	145.19	−37.43	10.3–11.3	531–2384	3.80	EBF	Forest	2006–2011
Warra	146.65	−43.10	10.0–10.2	1047–1291	1.74	EBF	Forest	2014–2015
Whroo	145.03	−36.67	15.4–16.1	912–491	0.94	WSA	Non-forest	2012–2016
Wombat	144.09	−37.42	11.0–12.0	694–1242	4.00	EBF	Forest	2010–2015
Yanco	146.29	−34.99	16.4–17.9	343–1119	0.64	CRO	Non-forest	2013–2016

IGBP biome types savanna (SAV), woody savanna (WSA), shrubland (OSH), grassland (GRA), evergreen broadleaf forest (EBF) and crop land (CRO). Ecosystem types defined in this study are non-forest or forest ecosystems (see Fig. 1). Ranges of mean annual surface air temperature (T_mean_ in °C) and annual precipitation (*P*_rcp_ in mm year^−1^) over the respective observation period (OP). Summary information about 23 eddy flux tower sites from the OzFlux network (http://www.ozflux.org.au, see Beringer *et al*.^13^). IGBP biome types savanna (SAV), woody savanna (WSA), shrubland (OSH), grassland (GRA), evergreen broadleaf forest (EBF) and crop land (CRO). Ecosystem types defined in this study are non-forest or forested ecosystems (see Fig. 1). Ranges of mean annual surface air temperature (T_mean_ in °C) and annual precipitation (*P*_rcp_ in mm year^−1^) over the respective observation period (OP). LAI is annual mean leaf area index derived from MODIS.

**Figure 3:**
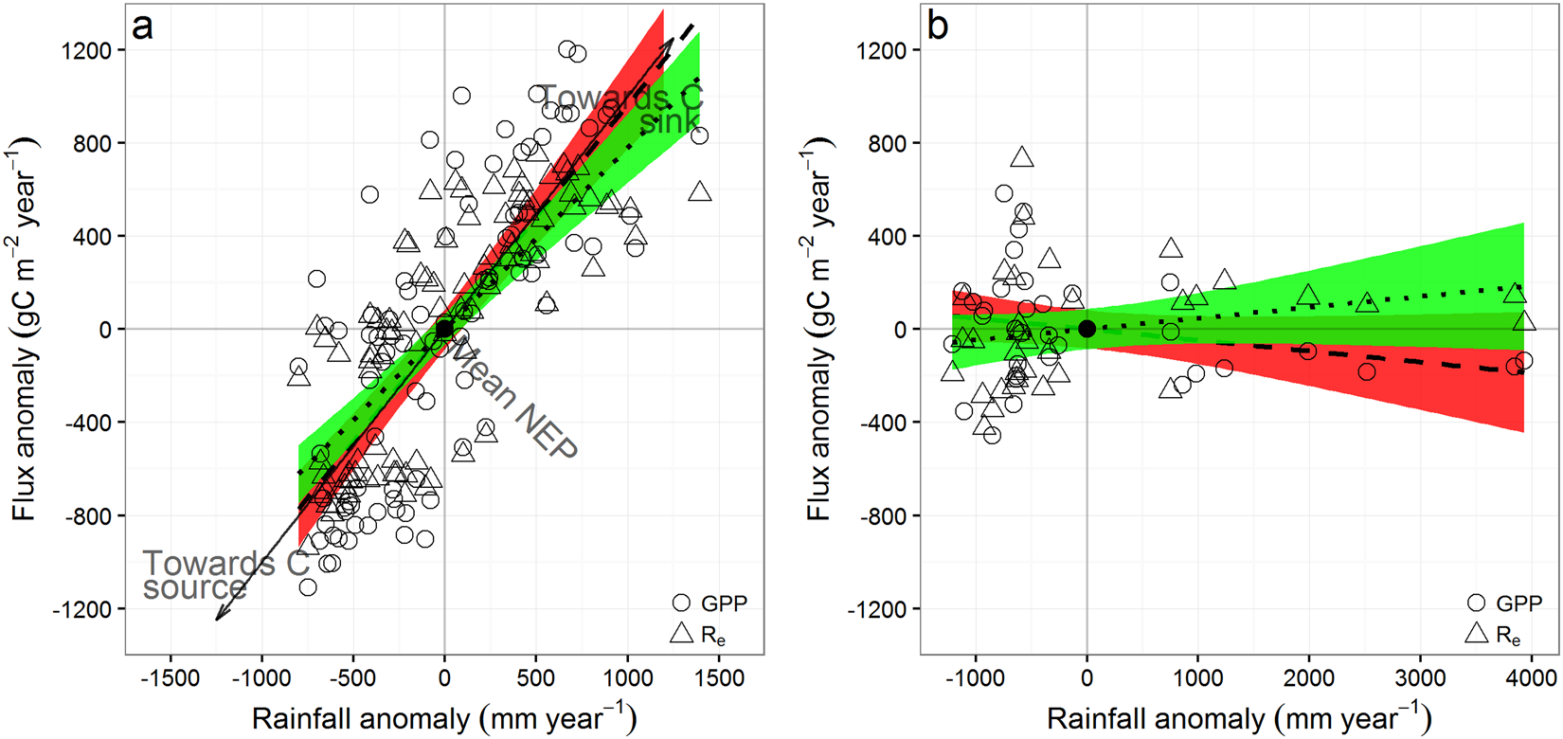
Responses of gross primary production (GPP) or ecosystem respiration (R_e_) anomalies to rainfall anomalies for non-forest (**a**) and forest (**b**) ecosystems in Australia. Open circles and triangles represent GPP and R_e_ anomalies, respectively. The dashed and dotted lines represent the best-fitted linear regressions between the anomalies of annual GPP or R_e_ and rainfall anomalies, and the red or green regions represent 95% confidence intervals. Ecosystems tended to be source when annual rainfall was below the multi-year mean, or a sink otherwise. Site measured rainfall were used in the analysis.

The different correlations among the component carbon fluxes between non-forest and forest ecosystems are important for identifying the different key driver of interannual variations of NEP. Among all climatic variables in Australia, coefficient of variation of annual rainfall is greatest. Both ΔGPP and ΔR_e_ were positively and significantly correlated with Δrainfall (*r* ^2^ = 0.58, *P* < 0.001 for ΔGPP and *r* ^2^ = 0.60, *P* < 0.001 for ΔR_e_, Fig. 3a) in non-forest ecosystems but not significantly in forest ecosystems. Sensitivity of GPP to rainfall anomalies (slope of linear regression equal to 0.96 gC m^−2^ mm^−1^ H_2_O) for the non-forest ecosystems was larger than that for R_e_ (0.78 gC m^−2^ mm^−1^H_2_O), although the difference was not statistically different (*P* = 0.09).

Because of the systematically greater sensitivity of ΔGPP to rainfall than ΔR_e_ to rainall and high correlation between ΔGPP and ΔR_e_ for non-forest ecosystem, ΔR_e_/ΔGPP is relatively conservative (0.79 ± 0.54), and ΔNEP is also found to increase significantly with an increase in rainfall for non-forest ecosystems (see Fig. 3a). Therefore the non-forest ecosystems are stronger carbon sink (more positive NEP) when annual rainfall is above the multi-year mean, (see Fig. 3a). For forest ecosystems, we found no significant correlation between ΔGPP, or ΔR_e_ or ΔNEP with rainfall (ΔGPP = −0.05ΔRainfall, *r* ^2^ = 0.04, *P* = 0.13 and ΔR_e_ = 0.05ΔRainfall, *r* ^2^ = 0.03, *P* = 0.16, see Fig. 3b), and the ratio of ΔR_e_/ΔGPP is quite variable (−0.34 ± 1.89), therefore NEP (carbon sink or source) was independent of inter-annual variation in rainfall.

### The response of canopy LAI to rainfall anomalies

Because of the high and positive correlation between GPP and R_e_, and GPP and NEP for the non-forest ecystems, we consider that R_e_ is largely limited by carbon substrate and that NEP is largely driven by the variation of GPP for non-forest ecosystems. To analyse the variation of GPP for both non-forest and forest ecosystems, we further decompose GPP into GPP per unit LAI and LAI to determine which component, GPP/LAI or LAI dominates the variation of GPP. For non-forest ecosystems by normalizing each variable within its range. That method was previously used to study NEP anomalies^1^. We found that GPP/LAI and LAI was not significantly correlated (*r* ^2^ = 0.009, *P* = 0.18), and variation of GPP was largely related to both LAI (with a slope of 0.88, *r* ^2^ = 0.75, *P* < 0.001) and GPP/LAI (with a slope of 0.86 and r^2^ = 0.33, *P* < 0.001, see Table 2). In contrast, neither GPP/LAI nor LAI *per se* were significantly correlated with GPP anomalies (*r* ^2^ = 0, *P* = 0.27 for GPP/LAI and *r* ^2^ = 0, *P* = 0.33 for LAI, *r* ^2^ = 0.78, *P* < 0.001 for GPP/LAI and LAI, see Table 2), as a result of the significant and negative correlation between GPP/LAI and LAI (with a slope of −0.84 and *r* ^2^ = 0.78, *P* < 0.001, see Table 2) for forest ecosystems. Furthermore, rainfall anomalies explained 49% of LAI anomalies for non-forest ecosystems (*P* < 0.001, see Table 2). Because the sensitivity of LAI to rainfall was large and significant for non-forest ecosystems (slope = 0.84, *P* < 0.001) but not significant in forest ecosystems (slope = 0.09, *P* = 0.53), we conclude that the main mechanism controlling inter-annual variations of GPP in non-forest ecosystems is the rainfall driven large variation in canopy LAI and to less extent in GPP/LAI.

**Table 2:** Statistics of the best-fitted linear regression between GPP anomalies and LAI or GPP/LAI anomalies and between GPP/LAI and LAI anomalies, and between LAI or GPP anomalies per unit of LAI (GPP/LAI) and rainfall anomalies.

Correlation	Ecosystem type	Slope	*r* ^2^	*P* value
GPP ~ LAI	Non-forest	0.88	0.75	<0.001
	Forest	0.19	0	0.33
GPP ~ GPP/LAI	Non-forest	0.86	0.33	<0.001
	Forest	0.22	0	0.27
GPP/LAI ~ LAI	Non-forest	0.09	0.009	0.18
	Forest	−0.84	0.78	<0.001
LAI ~ rainfall	Non-forest	0.84	0.49	<0.001
	Forest	0.09	−0.02	0.53
GPP/LAI ~ rainfall	Non-forest	0.27	0.1	<0.001
	Forest	−0.14	0	0.3

All variables (x) were normalised using the formula (x − x_n_)/(x_m_ − x_n_), where x_m_ and x_n_ represent the maximum and minimum values of the variable x. Statistics of the best-fitted linear regression between GPP anomalies and LAI or GPP/LAI anomalies, and between LAI or GPP anomalies per unit of LAI (GPP/LAI) and rainfall anomalies. All variables (x) were normalized using the formula (x − x_n_)/(x_m_ − x_n_), where x_m_ and x_n_ represent the maximum and minimum values of the variable x.

### Comparing the observed and simulated mechanisms by the TRENDY models

To quantify the contributions of GPP/LAI and LAI to the variance of GPP, we used the log-tranformation, ie log(GPP) = log(GPP/LAI) + log(LAI). Therefore var(log(GPP)) can be further decomposed into the contributions by the variations of log(GPP/LAI) and log(LAI) and their covariance using the observations from 23 flux towers in Australia or the four TRENDY^14^ model simulations for both non-forest and forest ecosystems in Australia.

**Figure 4:**
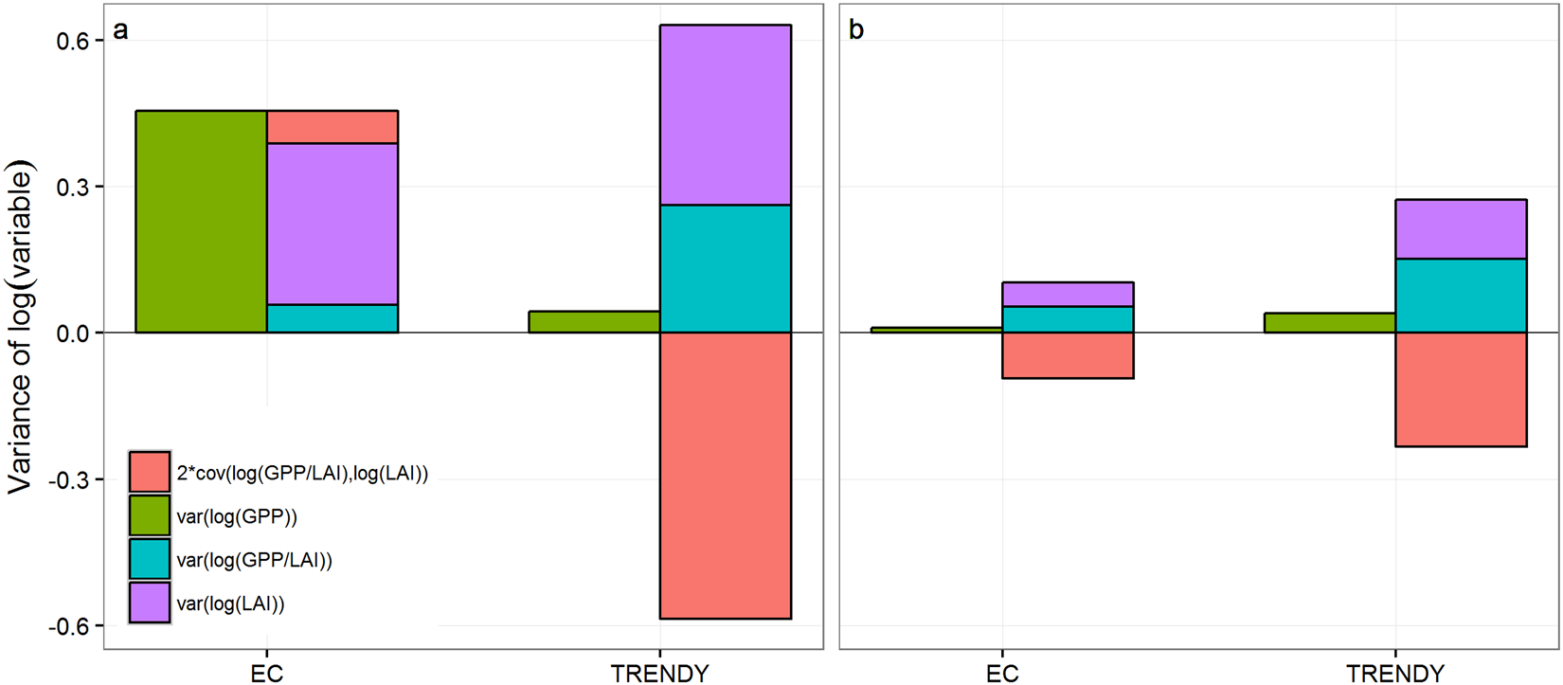
Comparisons of the variances of log-transformed GPP, LAI, GPP/LAI and the covariance between the latter two between measurements (EC) and the simulations by the TRENDY models (TRENDY) for non-forest (**a**) and forest ecosystems in Australia.

On average the TRENDY models failed to reproduce the dominant role of LAI in controlling GPP IAV for non-forest ecosystems (Fig. 4a) and overestimated the variances of log(LAI), log(GPP/LAI) and their covariance for forest ecosystems by more than 100% (Fig. 4b). Furthermore, the observed covariance between log(LAI) and log(GPP/LAI) was positive, and only contributed about 15% of the variance of log(GPP) in non-forest ecosystems, whereas the covariance of the modelled (log(GPP/LAI) and log(LAI) is negative and nearly as large as the total variance of log(GPP) and log(LAI) for the non-forest ecosystems (Fig. 4a). As a result, the variance of the modelled log(GPP) is only 9% of the variance of the observed log(GPP). The contribution of the covariance of the observed log(GPP/LAI) and log(LAI) to the variance of the observed log(GPP) is different, i.e. negative for non-forest ecosystems and positive for forest ecosystems. This observed difference between non-forest and forest ecosystem was not correctly simulated by the TRENDY models. (see Fig. 4).

## Discussion

Most Australian non-forest ecosystems are shrublands and savannas that together significantly contributed to the IAV of global land carbon uptake over the last three decades^1^. Recent studies demonstrated that the Australian non-forest ecosystems are well adapted to the climate conditions with highly variable rainfall^15,16^. This study has further demonstrated that it is the rapid response of canopy LAI, and to much less extent the response of GPP/LAI that is the dominant the large contribution of Australian non-forest ecosystems to global land sink IAV. It can be very difficult to quantify the contributions of GPP/LAI and LAI to the variance of GPP if GPP/LAI and LAI are strongly correlated, as for the forest ecosystems in this study. For non-forest ecosystems, the correlation between GPP/LAI and LAI is quite weak (r^2^ = 0.009, *p* = 0.18), then decomposing GPP as the product of GPP/LAI and LAI allows us to identify whether GPP/LAI or LAI dominate the variation of GPP.

The dominant role of rainfall in controlling GPP of non-forest ecosystems in Australia was consistent with previous studies on semiarid ecosystems in Africa^17–20^ and South America^21^. Rainfall IAV in non-forest ecosystems (mostly in semi-arid or arid regions) are typically proportionally larger than those experienced in forest ecosystems (in wet regions)^22^ and we have shown that this also is true at the global scale using Tropical Rainfall Measuring Mission (TRMM) data for 2001–2013 (Fig. S1). Large IAV of rainfall in Australia resuls from strong interactions amongst El Niño–southern oscillation, the Indian Ocean dipole and the southern annular mode^9,23,24^, and will likely intensity under future warmer conditions^25^. Therefore the non-forest ecosystems in Australia and other parts of the world will likely continue playing a significant role in the global carbon cycle and interannual variation in the growth rate of atmospheric CO_2_ concentration into the future.

In Australian non-forest ecosystems, both GPP and R_e_ were highly sensitive to rainfall, which drove the ecosystem towards being a carbon sink in wetter years, and conversely, a source in drier years. The non-forest ecosystems can respond to large rainfall events during dry period by rapidly initiating leaf flush and leaf expansion (hence increased LAI), and increasing soil N mineralisation to supply nutrients. As result, photosynthesis (GPP) is enhanced. Rainfall-induced increases in canopy LAI and GPP also increase ecosystem autotrophic respiration (both growth and maintenance respiration). Furthermore increases in available soil moisture and soil nutrients arising from increased soil mineralizationstimulate heterotrophic respiration (soil microbial respiration)^26^. This interpretation is clearly supported by the strong correlation between annual R_e_ and rainfall in the non-forest ecosystems (Fig. 2b), and is also consistent with observations globally^10–12^. A previous analysis of observations from 238 flux sites found that GPP was about 50% more sensitive to a drought event than R_e_^5^, and that difference is larger than the observed from the non-forest systems in this study. However, if only the flux data for OSH (open shrubland) are used, the difference in the responses of GPP and R_e_ to drought as estimated from the global dataset by Schwalm *et al*.^5^ is quite similar to our finding here. For forest ecosystems, Schwalm *et al*. found a very weak and no sensitivity of GPP or R_e_ to drought, which is also consistent with our results (Fig. 3b).

In Australia, non-forest ecosystems encompass almost all of the mesic savanna and shrubland ecosystems, and these ecosystems have higher levels of available N than forest ecosystems, but only when soil is wet^26^. This rainfall-driven fluctuation in available soil N, together with increased soil moisture during wet periods, result in a tight coupling between GPP, R_e_ and NEP. As a result, the NEP of the grassland component in savanna and shrubland ecosystems is highly dependent on rainfall^9^. In contrast, forest ecosystems in Australia occur in regions with less seasonally varying and higher rainfall and do not respond IAV of rainfall as strongly as non-forest ecosystem. In addition, forests have deep root systems which access deep soil moisture reserves and/or groundwater and hence maintain moderate LAI and GPP during relatively dry years^27–29^. Further, forests have multi-annual leaf life spans^30^, which might account for the lack of response to IAV in rainfall. That is why there was no significant correlation of rainfall with LAI, GPP nor NEP for the Australian forest ecosystems.

Canopy LAI is determined by not only climatic variables (e.g. rainfall) but alo likely the increasing atmospheric CO_2_. FACE (Free-Air CO_2_ Enrichment) experiments have found that canopy LAI increased under higher CO_2_ for some forest species^31^. Modelling studies also concluded that increase in CO_2_ reduced stomatal conductance and caused increase in GPP and LAI, particularly in water limited environments^32^. Limited by short time span, effects of CO_2_ change on canopy LAI and GPP were not considered in our analyses here, and will be taken into account in future studies. In addition, fire disturbance can be another important factor influencing Australian net ecosystem exchange and inter-annual variation at multiple scales from leaf to landscape^33^. While the significantly different responses of carbon fluxes to rainfall have been identified between forest and non-forest ecosystem in Australia, interaction of rainfall variation with other elements of the ecosystem (e.g. herbivory) and disturbance (e.g. fire) have not been explored here, and should be considered when accounting Australian terrestrial net biome exchange and their inter-annual variation^34^.

The four state-of-the-art process-based global land models from TRENDY did not correctly simulate the different responses of LAI to interannual variation of rainfall between forest and non-forest ecosystems, they were unable to predict correctly the dominated role of LAI in contronling GPP IAV for non-forest ecosystems, and overestimated the magnitudes of IAV of log-transformed GPP, LAI, GPP/LAI and the covariance between the latter two variables for Australian forest ecosystems. Importantly, the TRENDY model simulated similar covariance betweeon log(LAI) and log(GPP/LAI) across both non-forest and forest ecosystems, whereas the observed covariance are different in sign between these two types of ecosystems (Fig. 4). This suggests that state-of-the-art process-based ecosystem models as currently structured, need further improvement for forest ecosystems. Explaining the differential responses of ecosystem production to canopy dynamics and rainfall anomalies amongst non-forest and forest ecosystems should be targeted as a high priority in future model improvement, particularly when these models are used to project the trend and IAV in terrestrial ecosystem carbon status.

## Methods

### Eddy covariance flux observations

We used observations from 23 flux tower sites within the OzFlux network^13^ (http://www.ozflux.org.au). This dataset consists of 126 site-years data across most major ecosystems types in Australia. All flux data were gap-filled using an Artificial Neural Network (ANN) model^35^. An ANN model was also used to estimate daytime R_e_ from night-time observations of ecosystem respiration. GPP was calculated as the difference between NEP and R_e_, but these are not independently derived. Further information about the 23 flux towers is provided in Table 1. Original field-based flux data were at a half-hour time-step, and were aggregated to annual values f all correlation analysis.

### TRENDY simulations

Four models (Table 3), with available LAI and carbon fluxes (GPP, R_e_ and NEP) from the latest version of the TRENDY project^14^ were used in this study. The four models used were CABLE^36^, CLM^37^, LPJ^38^, and VISIT^39^ (Table 2). We used the S2 simulations wherever a time-invariant pre-industrial land use mask^40^ was applied.

**Table 3:** Four process-based models (LSMs) from the TRENDY project^14^.

Model name	Data years	Spatial resolution	LSMs	Source
CABLE	2001–2013	0.5° × 0.5°	yes	Wang *et al*.^36^
CLM	2001–2013	0.5° × 0.5°	yes	Lawrence *et al*.^37^
LPJ	2001–2013	0.5° × 0.5°	no	Sitch *et al*.^38^
VISIT	2001–2013	0.5° × 0.5°	no	Ito *et al*.^39^

Four process-based models from the TRENDY project^14^.

TRENDY model results were used to simulate how carbon fluxes of terrestrial ecosystems respond to changes in climate and atmospheric concentrations of CO_2_. All four models were operated at a spatial resolution of 0.5° × 0.5°, and covered the period 1901 to 2013. To match the period of flux tower measurements, model results during 2001–2013 were used in this study.

### Satellite datasets

LAI data were obtained from the MOD15A2.005 product of 0.05° × 0.05° spatial resolution and monthly time resolution (http://e4ftl01.cr.usgs.gov/MOLT/MOD15A2.005/), and aggregated to yearly value for analysis. A 3 km by 3 km window centred on the flux tower of each site was used to approximate the footprints of flux towers. Missing values of LAI were filled using the singular spectrum analysis (SSA) method^41^.

### Classification of ecosystems

The MODIS land cover type product (http://e4ftl01.cr.usgs.gov/MOTA/ MCD12C1.051) at a spatial resolution of 0.05° by 0.05° in 2012 was used for classifying the 14 sites into different ecosystem types. First, we classified Australian ecosystems into six biomes based on the MODIS IGBP land cover map for 2012. These six biomes are: evergreen broadleaf (EBF), cropland (CRO), grassland (GRA), savanna (SAV), woody savanna (WSA) and shrubland (OSH). Then all EBF were defined as forest ecosystems and non-EBF biomes were defined as non-forest ecosystems. Water bodies, wetland, urban and other built-up areas, bare or sparsely vegetated land areas were ignored. Our defined non-forest ecosystems (Type I and II, Fig. 1) had an 81% overlap area with those ecosystems in arid or semi-arid climatic zones as defined using the Köppen-Geiger method^42^ (Fig. 1).

### Climate data

Monthly rainfall data at a spatial resolution of 0.05° by 0.05° were obtained from the Australian Bureau of Meteorology (http://www.bom.gov.au/jsp/awap/). Annual rainfall was calculated as the sum of monthly rainfall.

## Electronic supplementary material

Supplementary Information
