# Supplementary material for: Responses of LAI to rainfall explain contrasting sensitivities to carbon uptake between forest and non-forest ecosystems in Australia
Source: Sci Rep. 2017 Sep 15;7:11720. doi: 10.1038/s41598-017-11063-w (PMC5601939; doi:10.1038/s41598-017-11063-w)
Supplement: Supplementary file 1 — Supplementary Information [file 41598_2017_11063_MOESM1_ESM.pdf]

Supplementary Materials

Responses of LAI to rainfall explain contrasting sensitivities to carbon uptake between forest and non-forest ecosystems in Australia

Longhui Li[1, *], Ying-Ping Wang[2], Jason Beringer[3], Hao Shi[1], James Cleverly[1], Lei Cheng[4], Derek Eamus[1], Alfredo Huete[1], Lindsay Hutley[5], Xingjie Lu[2], Shilong Piao[6], Lu Zhang[4], Yongqiang Zhang[4], Qiang Yu[1]

[1] School of Life Sciences, University of Technology Sydney, Australia

[2] Key Laboratory of Vegetation Restoration and Management of Degraded Ecosystems, South China Botanical Garden, Chinese Academy of Sciences, Guangzhou 510650, China and CSIRO Oceans and Atmosphere, PMB 1, Aspendale, Victoria 3195 Australia

[3] School of Earth and Environment, the University of Western Australia, Australia

[4] CSIRO, Land and Water, Canberra, Australia

[5] Research Institute for the Environment and Livelihoods, Charles Darwin University, Australia

[6] Peking University, Beijing, China

[*] Corresponding author:

Longhui Li, School of Life Sciences, University of Technology Sydney, NSW 2007, Australia; Email: lilhchn@gmail.com

This PDF file includes:

- **Figure S1**

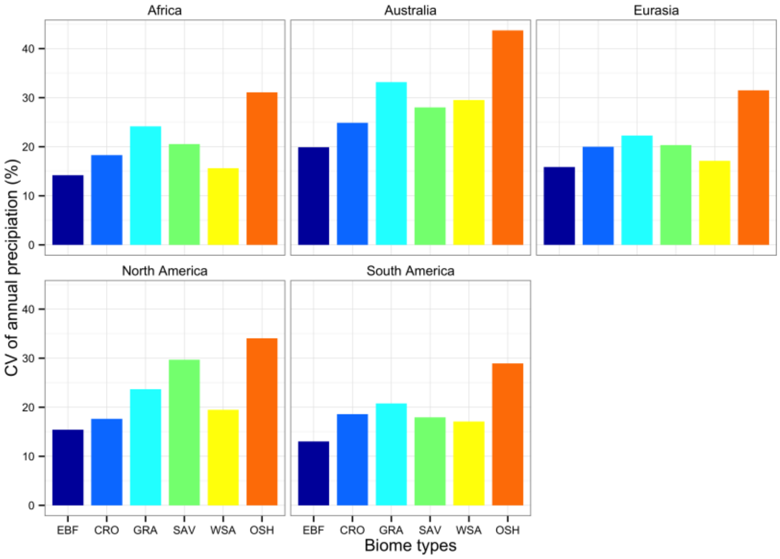

Figure S1 | Coefficient of variation (CV) of precipitation for different biome types over the five continents globally. Precipitation data were obtained from the Tropical Rainfall Measuring Mission (TRMM) 3B42 RT (http://pmm.nasa.gov/data-access/downloads/trmm). CV is defined as the ratio of the standard deviation to the mean during the period 2001-2013. EBF, CRO, GRA, SAV, WSA and OSH refer to evergreen broadleaf forest, cropland, grassland, savanna, woody savanna and shrubland.
